# Effects of SC-560 in Combination with Cisplatin or Taxol on Angiogenesis in Human Ovarian Cancer Xenografts

**DOI:** 10.3390/ijms151019265

**Published:** 2014-10-23

**Authors:** Wei Li, Liang Wan, Ling-Yun Zhai, Jane Wang

**Affiliations:** Department of Gynecology, Nanjing Medical University of Hangzhou Hospital, 261 Huansha Road, Hangzhou 310006, China; E-Mails: wanliang20069@126.com (L.W.); ZIyinhz@hotmail.com (L.-Y.Z.); wangjingjane@hotmail.com (J.W.)

**Keywords:** ovarian cancer, SC-560, cisplatin, taxol, angiogenesis

## Abstract

This study was designed to evaluate the effect of cyclooxygenase-1 (COX-1) inhibitor, SC-560, combined with cisplatin or taxol, on angiogenesis in human ovarian cancer xenografts. Mice were treated with intraperitoneal (i.p.) injections of SC-560 6 mg/kg/day, i.p. injections of cisplatin 3 mg/kg every other day and i.p. injections of taxol 20 mg/kg once a week for 21 days. Vascular endothelial growth factor (VEGF) mRNA levels were detected by reverse transcription-polymerase chain reaction (RT-PCR); microvessel density (MVD) was determined by immunohistochemistry; and prostaglandin E_2_ (PGE_2_) levels were determined using ELISA. Expression levels of VEGF mRNA and MVD in treatment groups were inhibited significantly when compared with the control group (*p* < 0.05 for all), and SC-560 combined with cisplatin displayed a greater reduction in the expression of VEGF and MVD than SC-560 or cisplatin alone (*p* < 0.05). SC-560 combined with taxol showed a greater inhibition on VEGF mRNA expression than SC-560 or taxol alone (*p* < 0.05). The level of PGE_2_ in treatment groups was significantly reduced when compared with the control group (*p* < 0.01 for all). These findings may indicate that cisplatin or taxol supplemented by SC-560 in human ovarian cancer xenografts enhances the inhibition effect of cisplatin or taxol alone on angiogenesis.

## 1. Introduction

Ovarian cancer is a common malignancy responsible for more deaths worldwide than any other malignancy of the female reproductive system [1]. Ovarian cancer growth is angiogenesis dependent and increased production of angiogenic growth factors such as vascular endothelial growth factor (VEGF) correlates with clinical stage, therapy efficacy, tumor metastasis and patient survival in human ovarian carcinoma [2]. At present, surgery, platinum and paclitaxel-based chemotherapy are still the main treatment methods for ovarian cancer [3]. Although 75%–80% of ovarian cancer patients can respond to chemotherapy at the initial stage, more than 80% of the patients that underwent chemotherapy may display drug resistance, even multi-drug resistance (MDR), ultimately, leading to the five year survival rate of only 30%, suggesting MDR is a common cause of the failure of chemotherapy in ovarian cancer [4]. Cisplatin is a platinum compound that was discovered in the 1960s and has been an important chemotherapeutic drug for the treatment of many cancers, including ovarian ones, as a single agent or in combination with other anticancer agents [5,6,7]. Taxol belongs to a family of microtubule-targeting drugs called the taxanes [8], which work by promoting assembly and stabilization of microtubules, thus preventing depolymerization. Taxanes are widely used to treat patients with lung, breast, stomach, endometrium or ovarian cancers [9]. The tolerance to taxol and cisplatin in ovarian cancer cells has been observed [10]; however the mechanisms of their resistance are not yet fully understood.

Cyclooxygenase (COX) is a key rate-limiting enzyme that catalyzes the biotransformation of arachidonic acid into prostaglandins and thromboxane, which mediate a range of physiological and pathophysiological responses [11]. The constitutively expressed isoform COX-1 is responsible for maintaining homeostasis and normal production of eicosanoids, whereas the inducible isoform COX-2 is implicated in the synthesis of prostanoids involved in acute and chronic inflammatory processes [12]. Studies have demonstrated that COX-2 up-regulates in a range of malignant neoplasms while the contribution of COX-1 remains undefined or controversial [13]. Dore *et al.* first documented that the epithelium covering the surface of the ovary, from which ovarian adenocarcinomas are believed to be derived, expressed abundant amounts of COX-1 [14]. A growing body of research focused on the up-regulation of COX-1 in many malignant tumors [15,16], especially in ovarian cancer as well as cell lines [17,18,19]. Moreover studies have demonstrated that COX-1 is over-expressed in various stages (onset and progression) of human epithelial ovarian cancers, where it controls the production of prostaglandins and promotes angiogenic growth factor production [13,17,19]. On the basis of these studies, it is concluded that COX-1 may contribute to carcinoma development in the ovary through stimulation of neovascularization. As a consequence, COX-1 might be an ideal target for theranostic investigations of human epithelial ovarian cancers [13].

Non-steroidal anti-inflammatory drugs (NSAIDs) are known to be inhibitors of the COXs and thus impede cancer growth primarily through blocking arachidonic acid metabolism by attenuating COX activity and reducing levels of prostaglandins. Ferrandina * et al.* found that COX-2 over-expression was associated with chemotherapy resistance [20], and its over-expression might reduce the efficacy of taxol [21]. In recent years, studies have reported the inhibitory effects of COX-2 inhibitors in combination with taxol on tumor growth [22,23]. Based on these studies, the combination of COX-2 selective inhibitor and taxol has already been used in phase II trials of some solid tumor treatment [24,25,26]. Numerous studies have shown that COX-1 is involved in the progression of ovarian carcinoma and that COX-1 selective inhibitors may inhibit tumor growth by inhibiting tumor angiogenesis [2,13,17,19,27]. However, studies of COX-1 inhibitors in combination with cisplatin or taxane on angiogenesis in human ovarian cancer xenografts have been rarely documented. In this study, using mice transplanted with a human ovarian cancer SKOV-3 cell lines as an experimental model system, we investigate the effects of SC-560, a selective COX-1 inhibitor, in combination with cisplatin or taxol on ovarian tumor growth and angiogenesis in a human ovarian cancer xenograft.

## 2. Results and Discussion

### 2.1. Inhibition of Ovarian Cancer Growth

**Figure 1:**
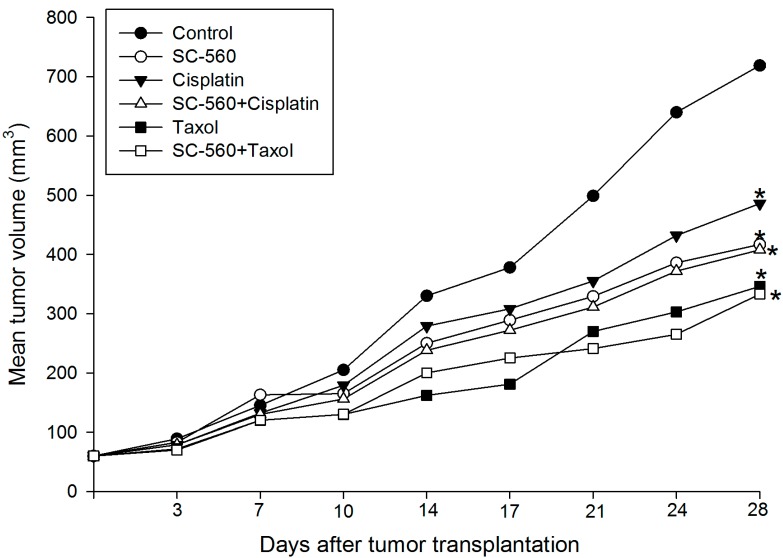
Effects of SC-560 combined with cisplatin or taxol on tumor growth *in vivo*. After 7 days had elapsed to allow for tumor establishment, mice were randomlyseparated into six groups (six mice in each group). Mice were treated with SC-560 (3 mg/kg twice a day), cisplatin (3 mg/kg every other day) and taxol (20 mg/kgonce a week) for 21 days. Average tumor volume of mice in all treatment groups was significantly different from control mice on day 28. * *p* < 0.05, compared with control.

When the tumors became visible (7 days after inoculation), mice were randomly separated into six groups (six mice in each group). SC-560 was administered by oral gavage at a dose of 3 mg/kg twice a day. Taxol was given by intraperitoneal (i.p.) injection at a dose of 20 mg/kg once a week. Cisplatin was administered by i.p. injection at a dose of 3 mg/kg every other day. Figure 1 shows the relative effect of SC-560 in combination with cisplatin or taxol on tumor growth. We observed that the tumor size increased throughout the period examined in the control group since the date of injection, whereas the average tumor size in all the drug-treated mice were significantly suppressed. For instance, on day 28, the mean tumor volume in control mice was 719 mm^3^. Under similar conditions, the mean tumor volume of the SC-560-treated group was 349 mm^3^ and cisplatin-treated group animals showed a mean tumor volume of 418 mm^3^. SC-560 was found to inhibit ovarian cancer growth better when compared with cisplatin in the experiment. And tumor growth was significantly reduced during the entire treatment period with SC-560. On day 28, the tumor size of mice in the SC-560, taxol and SC-560/taxol combination group was reduced by 44.67%, 54.48% and 55.35%, respectively, compared with the control mice. Moreover, the inhibitory effect observed in the SC-560, cisplatin, taxol and combination groups was all statistically significant compared with that of the control group (*p* < 0.05 for all).

### 2.2. Effect on Microvessel Density (MVD)

**Figure 2:**
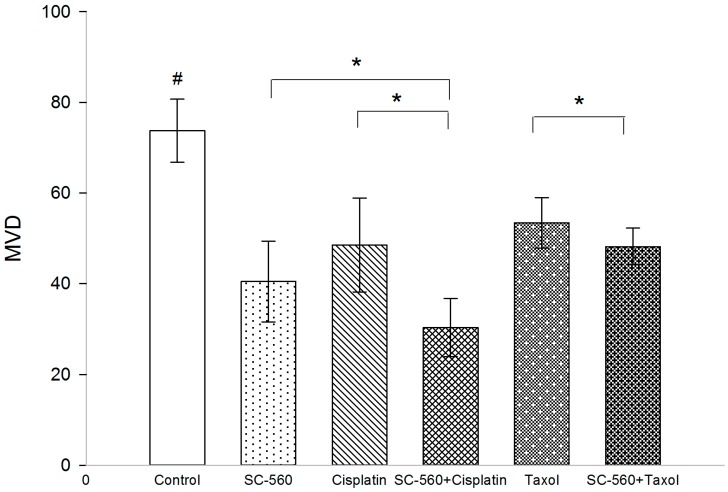
Effects of SC-560 combined with cisplatin or taxol on microvessel density (MVD) *in vivo*. MVD of control group compared withdrug-treated groups illustrated the significantly inhibitoryeffect of SC-560 in combination with cisplatin or taxol on tumor. ^#^ MVD of treatment groups compared with control group, *p* < 0.05 for all; * *p* < 0.05; error barsindicate standard error.

**Figure 3:**
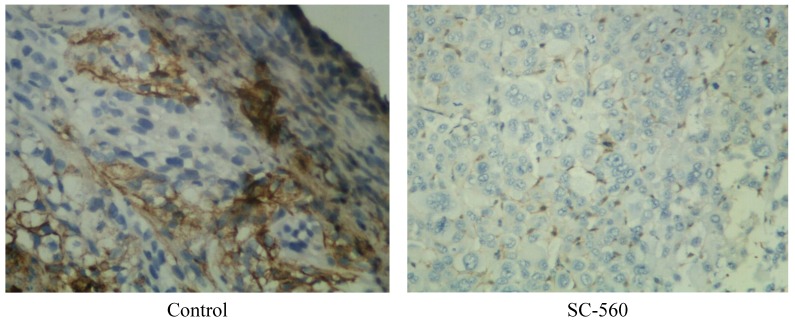
Effects of SC-560 on angiogenesis in SKOV-3 xenograft tumors.Representative pictures of CD34immunohistochemical staining of tumors. Magnification is 100×.

Immunohistochemical analysis of frozen tumor sections show a decrease in the number of CD34 positive microvessels in mice treated with SC-560, cisplatin, SC-560/cisplatin, taxol and SC-560/taxol (Figure 2). MVD in the treatment groups was 40.50 ± 8.92 (SC-560), 48.53 ± 10.37 (cisplatin), 30.30 ± 6.41 (SC-560/cisplatin), 53.43 ± 5.56 (taxol) and 48.20 ± 4.05 (SC-560/taxol), which was statistically significant compared with that of the control group (73.77 ± 6.94) (*p* < 0.05 for all). Sections from tumors grown in mice treated with SC-560 combined with cisplatin displayed a greater reduction in MVD compared with SC-560-treated group and cisplatin-treated group (*p* < 0.05). And SC-560 combined with taxol decrease the number of MVD to a greater extent when compared with the taxol-treated group (*p* < 0.05). Representative pictures of CD34 immunohistochemical staining of tumors show the effects of SC-560 on MVD in SKOV-3 xenograft tumors (Figure 3).

### 2.3. Effect on Prostaglandin E_2_ (PGE_2_) Production

**Figure 4:**
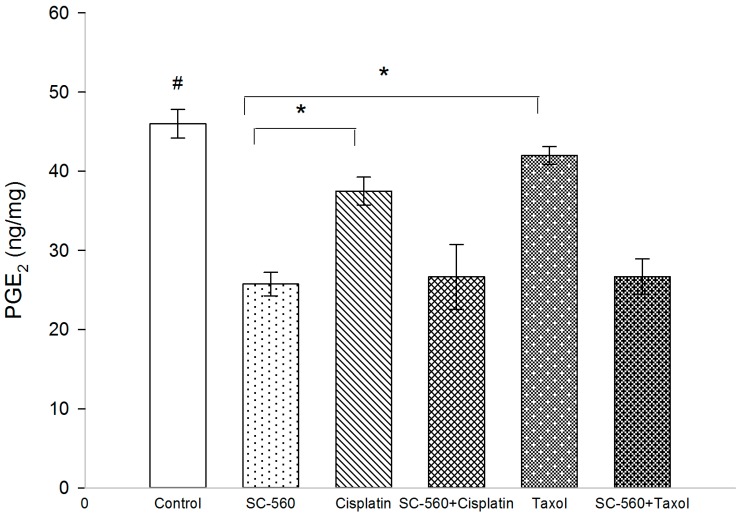
SC-560in combination with cisplatin or taxol affect prostaglandin E_2_ (PGE_2_) production. PGE_2_ of the control group compared withdrug-treated groups illustrated the significant inhibitoryeffect of SC-560 in combination with cisplatin or taxol on tumor. ^#^ PGE_2_ of treatment groups compared with control group, *p* < 0.01 for all; * *p* < 0.01; error barsindicate standard error.

To determine whether the anti-angiogenic activity of SC-560 in combination with cisplatin or taxol was due to the inhibition of PG synthesis, we tested prostaglandin E_2_ (PGE_2_) production by ELISA (Figure 4). The level of PGE_2_ in treatment groups was 25.75 ± 1.49 ng/mg (SC-560), 37.51 ± 1.76 ng/mg (cisplatin), 26.64 ± 4.11 ng/mg (SC-560/cisplatin), 42.00 ± 1.21 ng/mg (taxol) and 26.70 ± 2.25 ng/mg (SC-560/taxol), which was statistically significant compared with that of the control group (46.00 ± 1.81 ng/mg) (*p* < 0.01 for all). In addition, SC-560 showed a greater reduction in PGE_2_ production than cisplatin and taxol (*p* < 0.01). SC-560 to influence prostaglandin synthesis suggests that effects of SC-560 are mostly due to inhibition of COX-1 activity.

### 2.4. Correlation of PGE_2_ with MVD

**Figure 5:**
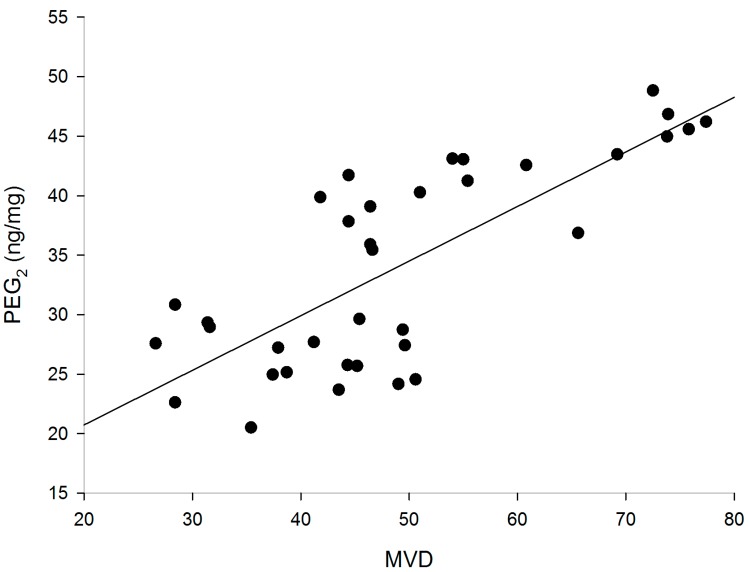
Correlation between the expressions of PGE_2_ andMVD (*r* = 0.764, *p* < 0.01).

Linear equations were created to show the correlation between MVD and PGE_2_ (Figure 5). The analysis revealed a positive correlation between the expressions of PGE_2_ and MVD (correlation coefficient, *r* = 0.764, *p* < 0.01).

### 2.5. Effect on Vascular Endothelial Growth Factor (VEGF) Production

**Figure 6:**
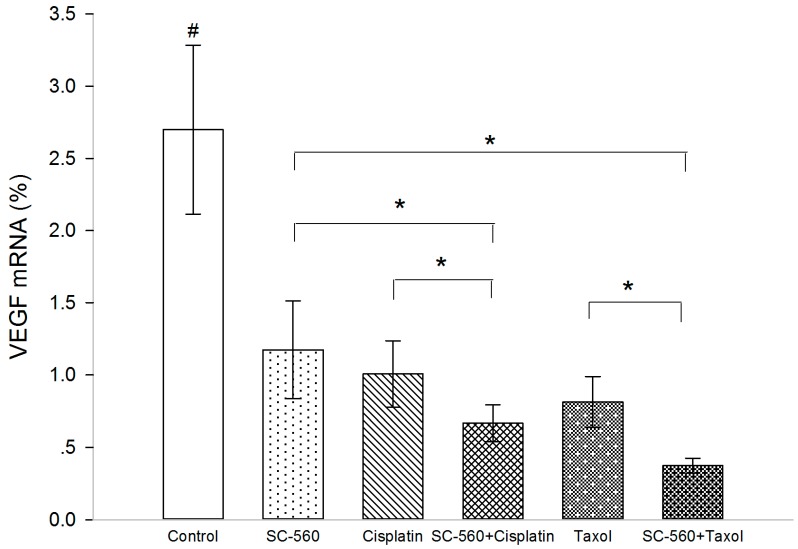
Effects of the drugs on the expression of *VEGF* mRNA.^#^ *VEGF* mRNA expression levels of treatment groups compared with control group, *p* < 0.05 for all; * *p* < 0.05; error bars indicate standard error.

In this experiment, we measured VEGF levels in xenograft tumors by real-time PCR analysis. Three molecular isoforms of VEGF were generated by alternative splicing, rendering proteins containing 189-, 165- and 121-amino acid residues. Real-time PCR analysis indicated the Δ*C*_t_ (cycle threshold, Δ*C*_t_ = *C*_t, selected gene_ − *C*_t, β-actin_) of VEGF in the three groups (Table 1). As shown in Figure 6, the expression levels of VEGF mRNA were significantly suppressed in the treatment groups (*p* < 0.05 for all). In addition, SC-560/cisplatin combination showed a greater inhibition on *VEGF* mRNA expression than SC-560 or cisplatin alone (*p* < 0.05). And SC-560/taxol combination therapy demonstrated a more synergistic effect than SC-560 or taxol alone on the inhibition of *VEGF* mRNA expression (*p* < 0.05).

**Table 1:** Δ*C*_t_ of VEGF in the six groups (control, SC-560, cisplatin, SC-560/cisplatin combination group, taxol and SC-560/taxol combination group) ^a^.

Group	VEGF 121	VEGF 165	VEGF 189
Control	6.94 ± 0.23	4.58 ± 0.26	6.34 ± 0.23
SC-560	7.13 ± 0.30	5.34 ± 0.30	6.54 ± 0.21
Cisplatin	7.71 ± 0.38	5.63 ± 0.51	7.14 ± 0.28
SC-560 + Cisplatin	8.16 ± 0.36	6.83 ± 0.37	8.27 ± 0.42
Taxol	7.87 ± 0.32	6.28 ± 0.49	7.79 ± 0.29
SC-560 + Taxol	9.14 ± 0.33	7.00 ± 0.48	8.99 ± 0.23

^a^ Molecular isoforms of VEGF were generated by alternative splicing, rendering proteins containing 189-, 165- and 121-amino acid residues. VEGF 189, 165 and 121 were routinely detected in this series of ovarian cancer.

### 2.6. Cyclooxygenase-1 (COX-1) Expression

**Figure 7:**
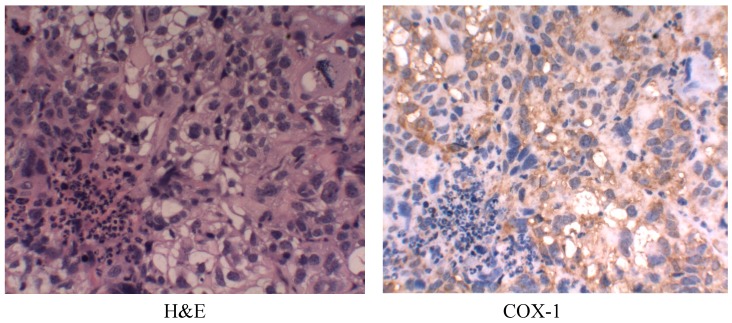
Immunohistochemicalanalysis of cyclooxygenase-1 (COX-1) expression in untreated tumor samples. Magnification is 100×.

The untreated tumors were analyzed for expression COX-1 (Figure 7).

### 2.7. Discussion

The genetic and molecular mechanisms underlying ovarian cancer remain largely unknown, and treatment options for patients with advanced disease are limited [19]. In 1998, Dore M. *et al.* [14] used immunohistochemistry to demonstrate strong expression of COX-1, not COX-2, protein in human ovarian cancer specimens. Since then, concerted efforts have been focused on COX-1 up-regulation and COX inhibition in many malignant neoplasms. Epidemiological evidence suggests that COX inhibition may reduce the risk of epithelial ovarian cancer by 40% or more [28]. COX-1-selective inhibitor was demonstrated to suppress tumor growth and metastasis in mice with established epithelial ovarian cancer and COX-1 inhibition procedures are now being examined for the preclinical treatment of tumor [13,17,19]. Our previous studies have demonstrated that SC-560 has chemopreventive properties and SC-560 combined with taxol show stronger growth-inhibitory effect [27,29]. Recently, Vladimir *et al.* has demonstrated that SC-560 suppress vasodilation of coronary vessels in a canine model and their findings suggest that endothelial COX-1 may be the primary COX isoform in the normal coronary arteries that mediates arachidonic acid induced vasodilation [30,31].

Debulking surgery followed by platinum–taxane based chemotherapy is the standard of care for patients with advanced stage ovarian cancer; however, despite an encouraging response rate of 65%–80% to first-line chemotherapy, most patients relapse with chemoresistant disease which has been the main cause for the failure of chemotherapy [32]. The most important mechanism responsible for the multi-drug resistance (MDR) phenotype is the over-expression of drug efflux transporter genes in cancer cells [33]. Combination chemotherapy is one of the strategies being used to overcome drug resistance. A number of studies revealed that taxol up-regulates the COX-2 level in tumor cells and enhances MDR1 expression and functional activity [21,34]. Okada *et al.* was the first to report that a COX-2 inhibitor prevented cisplatin-induced tumorigenesis in a mouse model [35]. Therefore, the addition of COX-2 inhibitors to taxol is widely used for antitumor treatment and the combination therapy has been used in phase II trials of solid tumor treatment [24,25,26]. However, research on cisplatin or taxol in combination with COX-1-selective inhibitors used for the chemotherapy of ovarian cancer has rarely been conducted.

Angiogenesis refers to the recruitment of new blood vessels and forms an essential component of the metastatic pathway. Numerous studies have indicated that angiogenesis is considered essential for tumor growth and the development of metastases [36,37,38]. Ovarian cancer is known to be highly vascular and is a primary cancer in which current anti-angiogenic therapies are being tested [39]. The present study has shown that SC-560 combined with cisplatin or taxol significantly suppressed the ovarian cancer growth in mice by inhibiting the number of MVD and the production of VEGF, thus ultimately impeding tumor angiogenesis. Previous studies have found that the expression of COX-1 leads to an increased expression of VEGF and that the inhibition of COX-1 reverses this response [17,27,40]. The ability of the COX-1 inhibitor to limit tumor growth may be through an indirect effect on tumor angiogenesis. The increase of MVD, an indirect marker of intense tumor vascularization, is known to be associated with both evolution of disease and survival. In our experiment, MVD in treatment groups displayed a reduction compared with control group and a combination of SC-560 and cisplatin demonstrated a synergistic effect on MVD. Also, our results had shown the expression levels of *VEGF* mRNA in treatment groups was suppressed and SC-560 combined with cisplatin or taxol therapy demonstrated synergistic effects. These results suggest that SC-560, when combined with cisplatin or taxol, enhances the anti-angiogenic effect of cisplatin or taxol. SC-560 may indirectly inhibit VEGF expression by inhibiting COX-1 expression and the decrease in tumor-associated VEGF may be a crucial mechanism in controlling angiogenesis. Recently, using the taxane-sensitive ovarian cancer cell line, SKOV-3, Lee *et al.* [41] reported that the combined treatment with paclitaxel and SC-560 promoted cytotoxicity in taxane-resistant ovarian cancers by suppressing *MDR1* gene and P-glycoprotein (P-gp), an ATP-binding cassette (ABC) transporter, expression. They reported that SC-560 significantly increased paclitaxel-induced cell death in taxane-resistant ovarian cancer cells in a prostaglandin- and COX-independent manner and their findings suggest that the COX-1 inhibitor can be a potent therapeutic tools not only as a drug sensitizer, but also as anti-angiogenic and pro-apoptotic agents. Their studies with taxane-sensitive ovarian cell lines were consistent with ours. Therefore, the potent anti-angiogenic activity of SC-560 combined with cisplatin or taxol seems to be the primary mechanism of action in the mice model of ovarian cancer.

In addition, the inhibition of COX-derived prostaglandin production was closely correlated with the decreased number of tumor MVD, and this result had been confirmed by Joarder * et al.* [42]. In our experiment, PGE_2_ levels were decreased in treatment groups compared with control mice and SC-560 showed a greater reduction in PGE_2_ production than cisplatin and taxol. The influence of SC-560 on PGE_2_ production suggests that effects of the COX-1 inhibitor are mostly due to inhibition of COX-1 expression; this result is in accordance with the study by Spinella *et al.* [43], who demonstrated that COX-1 inhibitor blocked PGE_2_ and VEGF release, indicating that COX-1 participates in PGE_2_ production. A previous study demonstrated that one of the mechanisms by which PGE_2_ supports tumor growth is by inducing the angiogenesis necessary to supply oxygen and nutrients to tumors >2 mm in diameter [44]. COX-derived PGs contribute to tumor growth in mouse models by inducing newly formed blood vessels that sustain tumor cell viability and growth. Thus, inhibition of COX derived prostaglandin production was closely associated with inhibition of angiogenesis, which is essential for tumor growth and the development of metastases. The present study demonstrated that SC-560 inhibited the production of PGE_2_, and that SC-560 enhanced the angiogenesis-inhibitory effect of cisplatin and taxol.

## 3. Experimental Section

### 3.1. Human Ovarian Tumors in Nude Mice

The human ovarian carcinoma cell lines SKOV3 in our experiments was chosen for its ability to mimic the progression of ovarian carcinoma when injected into *in vivo* mouse models and it could be well used to observe the antitumor effect [45,46]. The SKOV-3 cell line was purchased from China Type Culture Collection (Wuhan, China) and grown in the recommended media under standard condition. SKOV-3 cells were implanted subcutaneously in the dorsal skin (2 × 10^6^ cells) of female athymic nude mice (nu/nu, 7–8 weeks old). When the tumors became visible (7 days after inoculation), mice were randomly separated into six groups (six mice in each group): control, SC-560, cisplatin, SC-560/cisplatin, taxol and SC-560/taxol.

### 3.2. Dose and Administration of Drugs

SC-560 (Sigma Chemical Co., St. Louis, MO, USA) was administered by oral gavage at a dose of 3 mg/kg twice a day. Taxol (Bristol Myers Squibb SRL, Sermoneta, Italy) was given by intraperitoneal (i.p.) at a dose of 20 mg/kg once a week. The drugs were suspended in a 0.5 mL suspension of 5% methylcellulose (Sigma Chemical Co.) and 0.025% Tween 20 (Sigma Chemical Co.) Cisplatin was suspended in PBS (pH 7.2) by i.p. injection at a dose of 3 mg/kg every other day. Cisplatin was purchased from Haoshen Pharmaceutical Co. (Jiangshu, China). The dose of SC-560 was chosen for their specificity in inhibiting COX isotypes [47]. In a control group, mice were treated with physiological saline under similar conditions. The drugs or vehicle were administered over a period of 21 days, commencing 7 days after the tumors became palpable.

### 3.3. Measurement of Tumor Volume

The tumor dimensions were measured twice a week using a linear caliper, and tumor volume was calculated using the equation *V* (mm^3^) = *a* × *b*^2^/2, where *a* is the largest diameter and *b* is the smallest diameter [48]. Tumor growth was evaluated by the inhibition rate as assessed by the formula: *IR* = (*C* − *T*)/*C* × 100%, where *IR* is the mean inhibition rate, *T* is the mean tumor volume in the treatment group and *C* is the mean tumor volume in the control group. The animals were weighed weekly throughout the experiment. On day 28, all of the mice were sacrificed, and tumor tissue samples were collected and then fixed in 10% phosphate-buffered formalin solution for immunohistology or stored at 80 °C until analyzed. The tumor tissue samples were snap-frozen in liquid nitrogen before their storage at −80 °C.

### 3.4. Immunohistochemistry for MVD

Formalin-fixed paraffin-embedded tumor sections (6 μm) were subjected to immunostaining using CD34 antibodies (Santa Cruz Biotechnology, Dallas, TX, USA). Sections were deparaffinized and hydrated by sequential immersion in xylene and grade alcohol solutions. The sections were then incubated with 3% hydrogen peroxide in methanol solution for 34 min to block endogenous peroxidase activity. For antigen retrieval, slides were pressured in the pressure cooker for 2 × 10 min. For staining CD34, the sections were immersed in normal goat serum for 34 min. Immunohistochemical staining was performed using the streptavidin-biotin method. Microvessel density (MVD) was evaluated according to the method first described by Weidrer *et al.* [49]. The entire tumor section was first carefully scanned at low magnification with light microscopy (40×) to find the area that showed the most intense neovascularization. Because the immunohistochemistry of CD34 showed slight heterogeneity within the same tumor, the five most highly vascularized areas (hot spots) were selected in 200× magnification fields. The mean of five counts was calculated and used in statistical analysis.

### 3.5. Real-Time PCR

Total RNA was extracted using TRIzol reagents (Life Technologies, Shanghai, China), according to the manufacturer’s instructions. Isolated RNA was electrophoresed through 1.0% agarose-formaldehyde gels to verify the quality of the RNA. The first strand cDNA was generated by reverse transcription. After a sufficient amount of cDNA was obtained, we performed PCR amplification using a real-time PCR cycler (7500 ABI, ABI, New York, NY, USA). *VEGF* 189/165/121 RNAs were routinely detected in this series of ovarian cancer. The sequences of PCR primers were: *VEGF* (121), 5'-ACTCGGATGCCGACACGGGA-3' and 5'-CCTGGCCTTGCTTGCTCCCC-3'; *VEGF* (165), 5'-CCAGGATCCTCTGCCCGCCT-3' and 5'-GCGGCTTCCGGCACCTACAG-3'; *VEGF* (189), 5'-GGCAAAAGTTGCGAGCCGCC-3' and 5'-TGGATGGACCGGGAGCAGGG-3'; *β-actin*, 5'-GGGTGACGAGGCCCAGAGCA-3' and 5'-GGGGCCACACGCAGCTCATT-3'.

Amplification system included 50 μL of SYBRGreen Mix (32.5 μL), ddH_2_O (14.5 μL), cDNA (2 μL), forward primer (0.5 μL) and reverse primer (0.5 μL). The reaction conditions were as follows: Stage 1, 50 °C for 2.00 min (1 cycle); Stage 2, 95 °C for 5.00 min (1 cycle); Stage 3, 95 °C for 0.25 min followed by 60 °C for 0.75 min (40 cycles); Stage 4, 95 °C for 0.25 min firstly, then 60 °C for 1.00 min, and lastly, 95 °C for 0.25 min followed by 60 °C for 0.25 min (1 cycle).

The results of real-time PCR were analyzed by the DCT method: Δ*C*_t_ = *C*_t,selected gene_ − *C*_t,β-actin_, *RQ* (Relative Quantitation) = 2^−Δ*C*t^ × 100%. The results of real-time PCR were presented as the ratio between the selected genes and *β-actin* transcripts.

### 3.6. Determination of PGE_2_ Levels in Tumor Tissues

ELISAs: a PGE_2_ enzyme immunoassay kit (Cayman Chemical, Co., Ann Arbor, MI, USA) was used to quantify PGE_2_ concentrations in tumor extracts. PGE_2_ content in tumor tissue samples were determined using the method suggested by Trifan *et al.* [50]. Briefly, the tumor tissue samples were homogenized in prostaglandin extraction buffer (0.1 M phosphate, pH 7.4, containing 1 mM EDTA and 10 µM indomethacin) and incubated on wet ice for 30 min. The samples were centrifuged, and the supernatant was collected. A known volume of supernatant (typically 500 µL) was dried under nitrogen and resuspended in assay buffer and was analyzed as per the manufacturer’s recommendations (Cayman Chemical Co.). PGE_2_ values were expressed as microgram per milligram protein in the tissue samples.

### 3.7. Statistical Analysis

Statistical analysis was performed using SPSS software (SPSS version 17.0; SPSS, Chicago, IL, USA). Statistical significance between the control and treated groups was determined using the Student’s *t*-test. The experimental data were shown as the means ± standard error (SE). *p* < 0.05 was considered to indicate a statistically significant result.

## 4. Conclusions

Our data imply that cisplatin or taxol supplemented by SC-560 in the treatment of human ovarian cancer xenografts provides a synergistic inhibition effect compared to cisplatin or taxol alone on angiogenesis. However, whether COX-1 inhibitors combined with cisplatin or taxol therapy can be adopted as a new chemotherapy regimen in the treatment of ovarian cancer requires further intense research efforts.
